# Overexpression of *OsCASP1* Improves Calcium Tolerance in Rice

**DOI:** 10.3390/ijms22116002

**Published:** 2021-06-01

**Authors:** Zhigang Wang, Zhiwei Chen, Xiang Zhang, Qiuxing Wei, Yafeng Xin, Baolei Zhang, Fuhang Liu, Jixing Xia

**Affiliations:** State Key Laboratory for Conservation and Utilization of Subtropical Agro-Bioresources, College of Life Science and Technology, Guangxi University, Nanning 530004, China; 1808401019@st.gxu.edu.cn (Z.W.); 1808301008@st.gxu.edu.cn (Z.C.); 1708304025@st.gxu.edu.cn (X.Z.); 1808301051@st.gxu.edu.cn (Q.W.); 1908301066@st.gxu.edu.cn (Y.X.); 1908301074@st.gxu.edu.cn (B.Z.); 1708404002@st.gxu.edu.cn (F.L.)

**Keywords:** Casparian strip, suberin lamellae, apoplastic barrier, calcium, lignin

## Abstract

The Casparian strip domain protein 1 (*OsCASP1*) is necessary for the formation of the Casparian strip (CS) in the rice endodermis. It also controls Ca^2+^ transport to the stele. Here, we demonstrated that *OsCASP1* overexpression enhanced Ca tolerance in rice. Under normal conditions, *OsCASP1*-overexpressed lines showed similar concentrations of essential metals in the roots and shoots compared to the wild type, while under high Ca conditions, Ca in the roots, shoots, and xylem sap of the *OsCASP1*-overexpressed lines was significantly decreased. This did not apply to other essential metals. Ca-inhibited growth was significantly alleviated in the *OsCASP1*-overexpressed lines. Furthermore, *OsCASP1* overexpression resulted in earlier formation of both the CS and functional apoplastic barrier in the endodermis but did not induce ectopic CS formation in non-endodermal cell layers and affect suberin accumulation in the endodermis. These results indicate that the overexpression of *OsCASP1* promotes CS formation in endodermal cells and inhibits Ca^2+^ transport by the apoplastic pathway, restricting Ca accumulation in the roots and shoots under high Ca conditions. Taken together, the results suggest that *OsCASP1* overexpression is an effective way to improve rice adaptation to high Ca environments.

## 1. Introduction

Calcium (Ca) is one of the most abundant metal elements in soil, which normally contains large amounts of exchangeable Ca (300–5000 ppm) [1]. It is an essential macronutrient for plant growth and is involved in many biological processes, including cell division and expansion, structural integrity, and cellular signaling [2,3,4,5,6]. However, high cytosolic Ca^2+^ concentrations are cytotoxic [7,8]. Excessive Ca^2+^ restricts plant growth in calcareous soils [7]. Therefore, optimal Ca concentrations are required for normal plant growth and development.

Divalent cation Ca^2+^ in the soil solution are taken up by the root system and radially delivered to the xylem by apoplastic and symplastic pathways [9,10,11]. In roots, two endodermal barriers, Casparian strip (CS) and suberin lamellae, are considered to limit the apoplastic flow of Ca across the endodermis into the xylem. These two barriers develop gradually during the root differentiation [12,13]. At the primary developmental stage of endodermis, CS formation is initiated at a few millimeters from the root apex. After the CS formation and maturation in the root differentiation zone, suberin lamellae are deposited between the primary cell wall and the plasma membrane surrounding endodermal cells. When the CS located in the anticlinal cell wall between endodermal cells is completely formed, transport of Ca^2+^ to the xylem must use a symplastic pathway. In this case, the apoplastic Ca^2+^ diffuses into the cytoplasm of the endodermal cells through Ca channels in the plasma membrane [10]. Subsequently, Ca^2+^ is exported from the symplast of the endodermal cells into the stele by Ca^2+^ transporters such as Ca^2+^-ATPases [10]. When suberin lamellae cover the endodermal cells, direct Ca^2+^ uptake from the apoplast into the endodermis is prevented, and the Ca^2+^ must be taken up into the symplast from the outer cell layers [10]. However, the precise roles of these two endodermal barriers in regulating Ca^2+^ transport into the xylem for transfer to the shoot are poorly understood.

Interestingly, in *Arabidopsis*, several CS mutants such as *casp1, casp3*, *esb1*, *myb36*, and *lotr1* displayed similar shoot ionomic alterations, including low Ca accumulation [14,15,16]. It was proposed that an increase in endodermal suberization in these mutants inhibited Ca^2+^ transport into the xylem by the symplastic pathway, leading to the reduced Ca concentration in the shoots [15,16]. Rice differs from *Arabidopsis* in having two CSs in the exodermis and endodermis, respectively [17]. Apoplastic Ca^2+^ uptake may occur largely in the root tip where the CS is formed in the endodermis but not the exodermis [18]. A recent report shows that *OsCASP1* is only involved in CS formation in the root endodermis [18]. In *oscasp1*, defective endodermal CS formation resulted in uncontrolled apoplastic Ca^2+^ transport to the stele, which is prevented by the CS in the wild type, resulting in the over-accumulation of Ca in the shoot [18]. Therefore, the role of the CS in controlling Ca accumulation in shoots differs between *Arabidopsis* and rice.

To further investigate the role of *OsCASP1* in rice CS formation and Ca uptake, in this study, we generated *OsCASP1* overexpression transgenic lines and characterized them. We found that *OsCASP1* overexpression induced early formation of the CS in the root tip and selectively reduced Ca accumulation in the root and shoot under high Ca conditions. Furthermore, the overexpression lines enhanced rice tolerance to excess Ca. This work enriches our understanding of the interrelationships between CS formation, Ca uptake, and Ca tolerance in rice.

## 2. Results

### 2.1. Expression of OsCASP1 in Transgenic Lines

**Figure 1:**
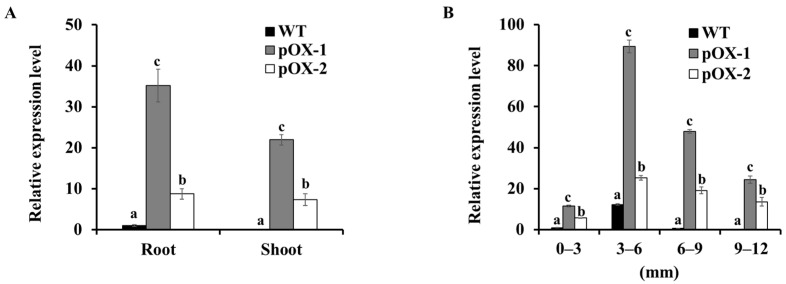
*OsCASP1* expression in different tissues of wild-type plants (WT) and *OsCASP1*-overexpressing rice lines (pOX-1 and pOX-2). (**A**) Expression of *OsCASP1* in roots and shoots. Expression relative to the wild-type root expression is shown. (**B**) Spatial *OsCASP1* expression in roots. Expression relative to the wild-type root expression (0–3 mm) is shown. RNA was extracted from different root parts (0 to 3, 3 to 6, 6 to 9, and 9 to 12 mm from the root tip) of 5-day-old seedlings. *Histone H3* was used as an internal standard. Data represent means ± SD (*n* = 3). Different letters indicate significant differences (Tukey test, *p* < 0.05).

*OsCASP1* was overexpressed in rice plants under the control of the maize ubiquitin promoter. Two independent transgenic lines (pOX-1 and pOX-2) were produced for subsequent experiments. RT-qPCR analysis showed significantly higher *OsCASP1* expression in both the roots and shoots of the two transgenic lines than in the wild-type rice (WT) (Figure 1A), ranging from a 10 to 35-fold increase. Furthermore, a spatial analysis revealed higher *OsCASP1* expression in the different root segments of the transgenic lines compared with the WT (Figure 1B).

### 2.2. The Role of OsCASP1 in Ca Tolerance

**Figure 2:**
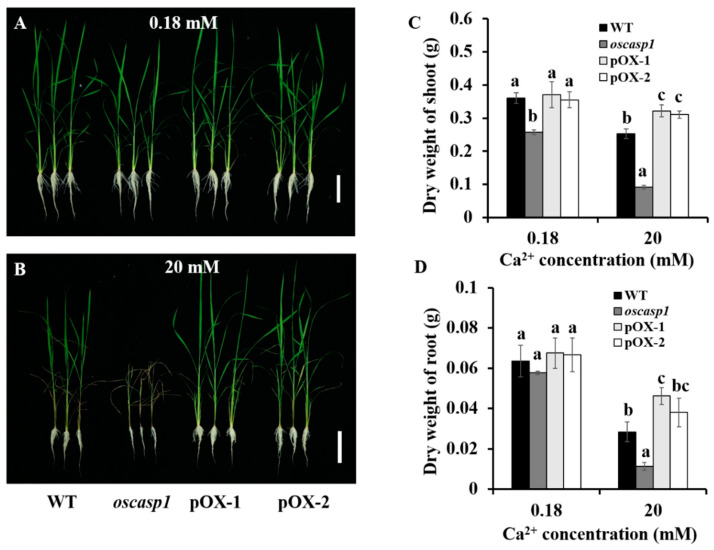
Growth of *OsCASP1*-overexpressing and wild-type rice seedlings. (**A**) Growth under the control condition (0.18 mM CaCl_2_). (**B**) Growth with high Ca (20 mM CaCl_2_). (**C**) Dry weights of shoots. (**D**) Dry weights of roots. Two-week-old seedlings of the wild type (WT), *oscasp1*, and two *OsCASP1*-overexpressing lines (pOX-1 and pOX-2) were grown in half-strength Kimura B solution with 20 mM CaCl_2_. After 12 d, plants were photographed and sampled. Bar = 10 cm. Data are mean ± SD; *n* = 3. Different letters indicate significant differences (Tukey test, *p* < 0.05).

Two pOX lines, the knockout line (*oscasp1-1*) and the WT Nipponbare, were used to measure phenotypic differences under normal (0.18 mM CaCl_2_) and high Ca^2+^ (20 mM CaCl_2_) conditions. Under normal conditions, similar growth was observed between the overexpressed lines and the WT, while shoot growth in the *oscasp1-1* knockout line was slightly retarded (Figure 2A). However, under high Ca^2+^ conditions, significant differences in plant growth were observed among the WT, *oscasp1-1*, and the pOX lines (Figure 2B). The leaves of both WT and the mutant showed evidence of necrosis. Moreover, growth in the mutant line was more seriously retarded than in the WT, with most of the leaves dry and wilted (Figure 2B). In contrast, the leaves in the two pOX lines appeared largely normal and unaffected by the high Ca concentration (Figure 2B). Correspondingly, the dry weights of the shoots and roots from these lines were significantly higher than those of the WT and knockout lines under high Ca^2+^ conditions (Figure 2C,D). Taken together, the phenotypic data indicated that the overexpression of *OsCASP1* can increase Ca tolerance in rice.

### 2.3. Mineral Analysis of OsCASP1 Overexpression Lines

**Figure 3:**
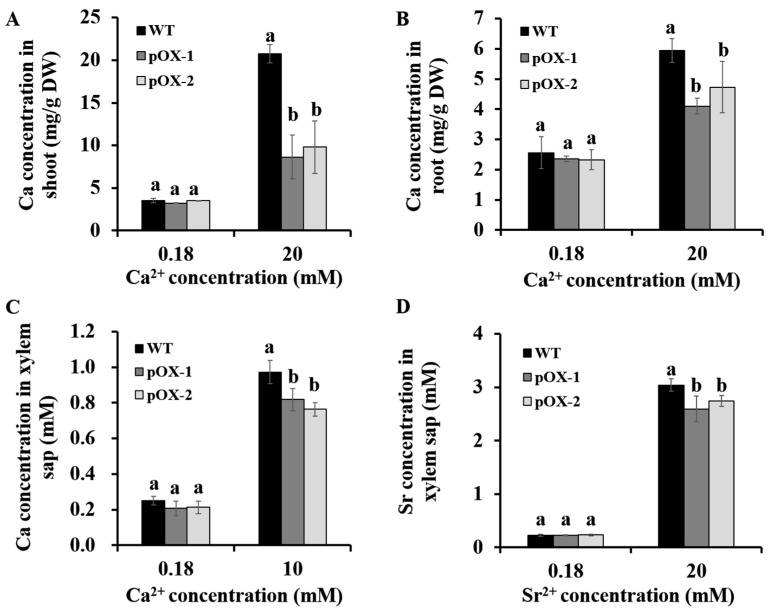
Ca concentrations in *OsCASP1*-overexpressing lines (pOX-1, pOX-2) and wild-type rice plants (WT). (**A**) Ca concentration in shoots. (**B**) Ca concentration in roots. Two-week-old seedlings were grown in nutrient solutions containing 0.18 or 20 mM CaCl_2_ for 12 d. (**C**) Ca concentration in xylem sap. Xylem sap was collected from plants exposed to different Ca^2+^ concentrations (0.18 and 10 mM) for 6 h. (**D**) Sr concentration in xylem sap. Xylem sap was collected from plants exposed to different Sr^2+^ concentrations (0.18 and 20 mM) for 6 h. Data are means ± SD (*n* = 3). Different letters indicate significant differences (Tukey test, *p* < 0.05).

No differences were observed in tissue mineral concentrations between the pOX and WT lines under normal conditions (Figure 3A,B) (Supplementary Figures S2A,C and S3A,C). Under conditions of high Ca^2+^, the Ca levels in both shoots and roots of the pOX lines were significantly lower than in the WT shoots (Figure 3A,B). No differences were observed in the concentrations of other metals, including K, Mg, Cu, and Zn, between the plants (Supplementary Figures S2B,D and S3B,D). These results indicated that overexpression of *OsCASP1* resulted in reduced Ca accumulation in both roots and shoots under high Ca conditions.

To determine the reason for the low Ca accumulation in the pOX shoots, we compared Ca and Sr concentrations in the xylem sap of the plants under different Ca or Sr treatments. Sr has similar chemical properties to Ca and was used as a tracer of apoplastic transport from root to shoot. Under normal conditions, the Ca and Sr concentrations in the xylem sap were similar between the pOX and WT plants (Figure 3C,D). However, under high Ca or Sr treatment, the xylem sap in the pOX lines showed significantly lower Ca/Sr concentrations than in the WT rice (Figure 3C,D), suggesting that the overexpression of *OsCASP1* inhibited apoplastic Ca^2+^ transport to the shoot, resulting in low Ca accumulation in the pOX shoots under high Ca conditions.

### 2.4. Effect of OsCASP1 Overexpression on CS Formation

**Figure 4:**
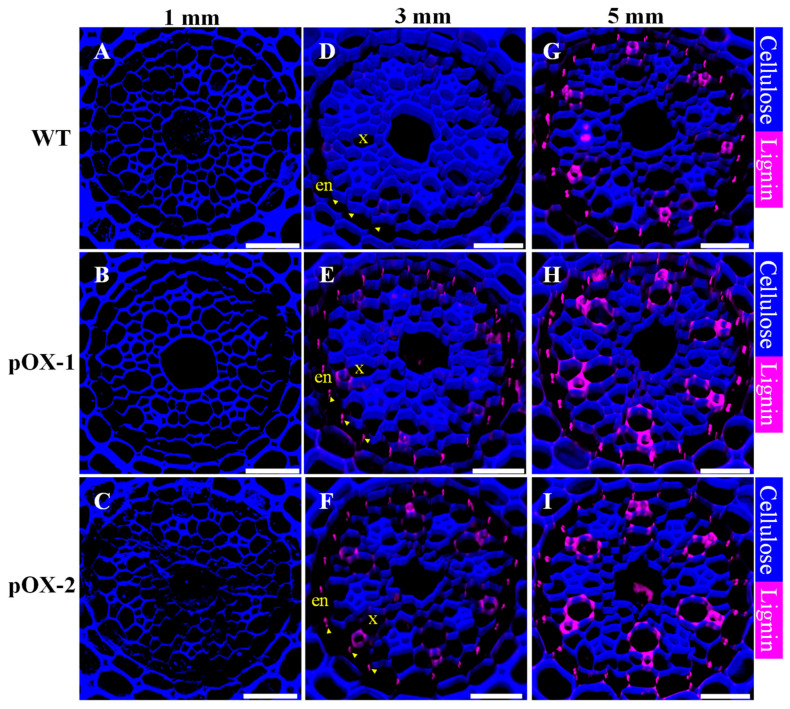
Casparian strip formation in *OsCASP1*-overexpressing lines (pOX-1, pOX-2) and wild-type rice plants (WT). (**A**–**I**) Casparian strip formation in the wild type and two *OsCASP1*-overexpressing lines. Lignin staining of root sections (1, 3, and 5 mm from the apex) of wild-type (**A**,**D**,**G**), pOX-1 (**B**,**E**,**H**), and pOX-2 (**C**,**F**,**I**). Magenta color represents lignin and blue represents cellulose. The sections were stained with 0.2% Basic Fuchsin and 0.1% Calcofluor White for 30 min, respectively. Five roots of each line (WT, pOX-1, and pOX-2) are examined and show the same phenotype. The Casparian strip is indicated by arrowheads. en, endodermis; x, xylem vessel. Scale bars, 20 μm.

To investigate the effect of *OsCASP1* overexpression on CS formation, we investigated lignification in the roots as lignin is a main component of the CS. Histochemical staining of sections at 1 and 3 mm from the root tip showed no lignin deposition in WT endodermal cells (Figure 4A,D) (Supplementary Figure S4A,D), which is consistent with previous results [18]. In contrast, in the two *OsCASP1*-overexpressed lines, the endodermal cells exhibited restricted, dot-like lignin staining (Figure 4B,C,E,F) (Supplementary Figure S4B,C,E,F). However, further from the tip (5 mm), the lignin deposition pattern was similar to that of the WT (Figure 4G–I) (Supplementary Figure S4G–I). This indicates that *OsCASP1* overexpression induced early CS formation in the root tip region.

**Figure 5:**
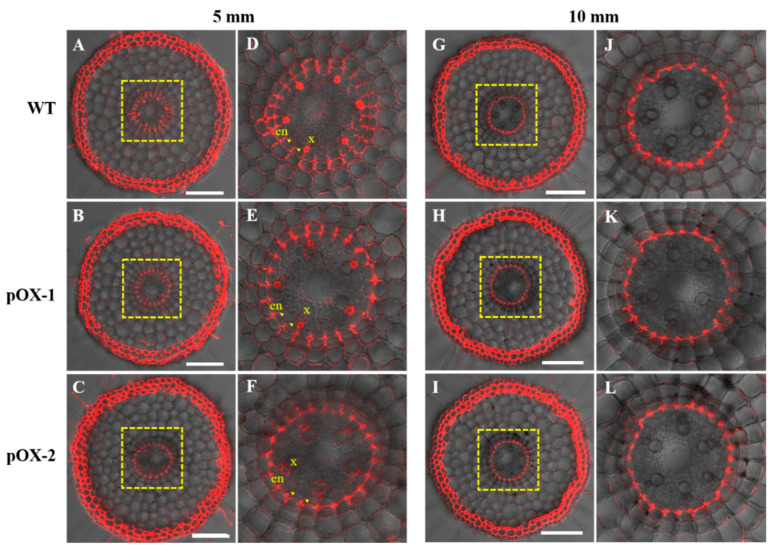
Casparian strip functionality of *OsCASP1*-overexpressing lines (pOX-1, pOX-2) and wild-type rice plants (WT) evaluated by PI penetration. PI penetration in the roots of wild-type (**A**,**D**,**G**,**J**), pOX-1 (**B**,**E**,**H**,**K**), and pOX-2 (**C**,**F**,**I**,**L**) plants at 5 mm (**A**–**F**) and 10 mm (**G**–**L**) from the root apex. Five-day-old seedlings were exposed to PI in the dark for 40 min. (**D**–**F**,**J**–**L**) are magnified images of the yellow boxed area in (**A**–**C**,**G**–**I**) respectively. Five roots of each line (WT, pOX-1, and pOX-2) are examined and show the same phenotype. The Casparian strip is indicated by arrowheads. en, endodermis; x, xylem vessel. Scale bars, 100 μm.

Then, we investigated diffusion of the apoplastic tracer PI into the stele as a measure of the functionality of the endodermal apoplastic barrier [15]. Less PI penetrated the stele of the overexpressing lines at 5 mm from the root apex than in the WT (Figure 5A–F). However, at the start of the root elongation zone (1 cm from the root apex), no PI penetration was observed in any of the lines (Figure 5G–L). These results suggested that the diffusional barrier formation in the overexpressing lines was developed earlier compared with the WT.

### 2.5. Effect of OsCASP1 Overexpression on Suberin Accumulation

**Figure 6:**
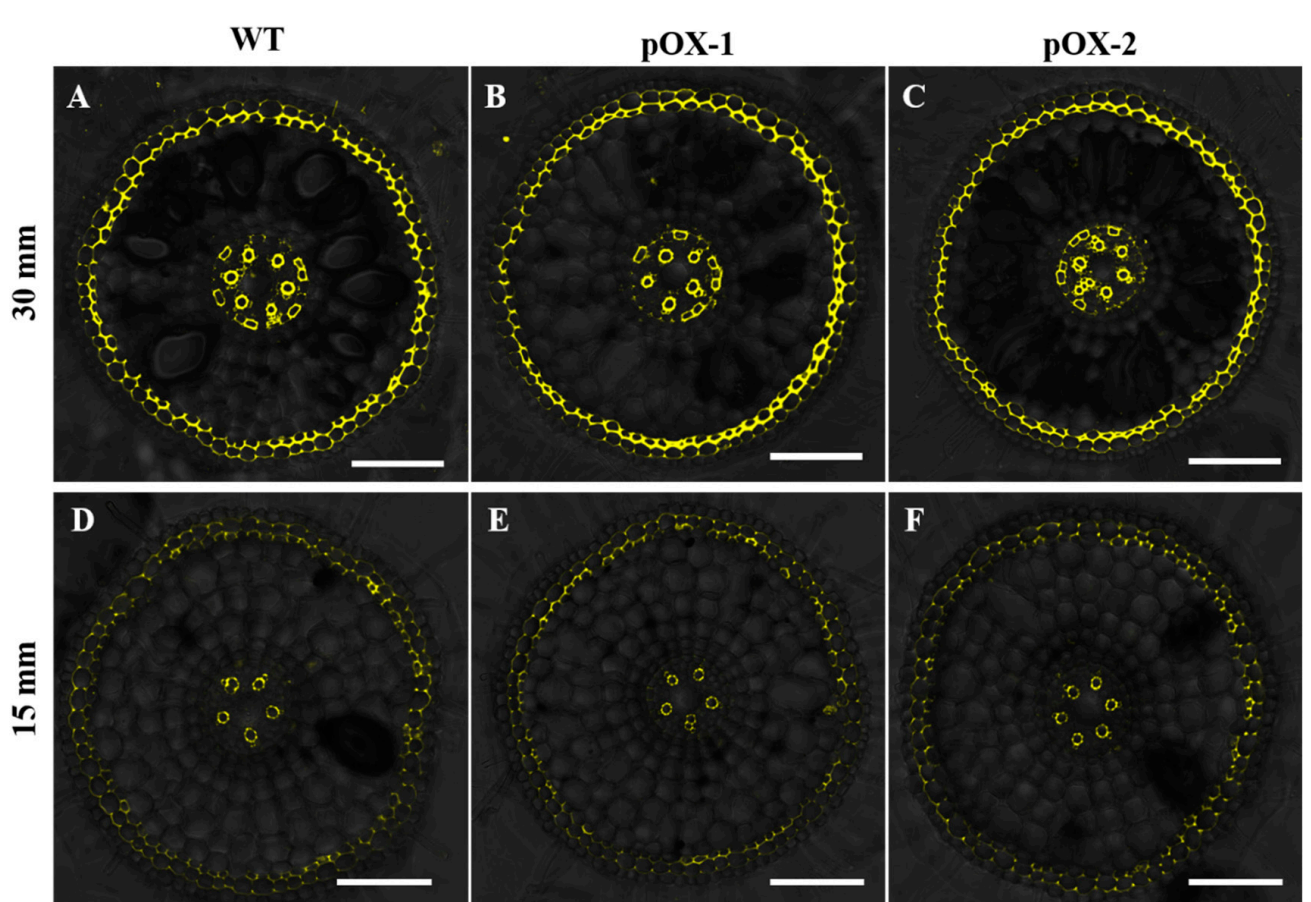
Suberin staining of the *OsCASP1*-overexpressing lines (pOX-1, pOX-2) and wild-type rice plants (WT). Suberin staining of roots (15 and 30 mm from the apex) from WT (**A**,**D**), pOX-1 (**B**,**E**), and pOX-2 (**C**,**F**). Root cross sections were stained with 0.01% (*w*/*v*) Fluorol Yellow 088 in lactic acid at 70 °C for 30 min. Five roots of each line (WT, pOX-1, and pOX-2) are examined and show the same phenotype. Yellow color shows suberin signal. Bar = 100 µm.

To test whether overexpression of *OsCASP1* affected suberin accumulation in the endodermal cells, we investigated suberin deposition in different parts of the roots. Minimal suberin accumulation was observed at 15 mm from the root tip in both the WT and pOX lines (Figure 6D,F), while at 30 mm from the tip, suberin was visible in several endodermal cells (Figure 6A–C). However, there was no difference in suberin accumulation in the endodermis between the WT and two pOX lines (Figure 6A–C). These results indicated that the overexpression of *OsCASP1* did not affect suberin accumulation in the endodermis.

## 3. Discussion

The CS functions as a physical barrier that prevents unfavorable inflow and backflow of nutrients between the soil and the stele and is considered to play an important role in environmental adaptation in plants [12,13]. For example, the *Arabidopsis* CS mutant *gso1/sgn3* displayed a severe potassium deficiency phenotype under low potassium conditions [19]. Another *Arabidopsis* CS mutant *lotr1* showed hypersensitivity to low Ca^2+^ conditions [16]. Recently, the mutation of both CS integrity factor1 (CIF1) and CIF2 in *Arabidopsis* produced noticeable growth defects under excess iron conditions [20,21]. More recently, the *oscasp1* mutant with defective CS formation showed hypersensitivity to high Ca conditions in rice [18]. However, the role of the CS in plant adaptation to the growth environment remains unclear. Rice is usually cultivated under flooded conditions, where Ca^2+^ concentration in soil solution is high due to the dissolution of calcium carbonate in acid soils. Especially, the soil of karst areas contains a higher Ca^2+^ concentration [22]. In the present study, we reported that the overexpression of *OsCASP1* can improve rice adaptation to high Ca stress. The expression of *OsCASP1* in the pOX lines was significantly enhanced in all tissues, including the root tip, compared with the WT. Under normal conditions, the concentrations of various essential metals in the roots and shoots were found to be similar between the WT and pOX lines. Under high Ca conditions, the Ca^2+^ concentration in the roots, shoots, and xylem sap of the pOX lines was significantly decreased in contrast to other essential metals. Furthermore, Ca-inhibited growth was significantly alleviated in the pOX lines. In addition, *OsCASP1* overexpression resulted in earlier CS formation in the endodermis but did not affect suberin accumulation. This early CS formation inhibited apoplastic Ca transport, resulting in less Ca^2+^ in the shoot. These results indicated that the overexpression of *OsCASP1* can reduce Ca accumulation in the shoots and enhance Ca tolerance in rice.

In *Arabidopsis*, localization of AtCASPs at the CS membrane domain (CSD) initiates CS formation [23]. Subsequently, other proteins such as enhanced suberin 1 (ESB1) and peroxidase 64 (PER64) are recruited to the CSD for lignin biosynthesis and deposition by the AtCASPs [24,25]. OsCASP1 functions in a similar manner to the AtCASPs, forming a transmembrane scaffold that mediates CS formation in the endodermis [18]. In the WT, we observed an initiation of CS formation at 5 mm from the root tip in conjunction with high *OsCASP1* expression (Figure 4A,D). In the pOX lines, *OsCASP1* was highly expressed throughout the root. As a result, the start site of lignin deposition in the endodermis in the pOX lines was closer to the root apex relative to the WT (Figure 4B,C,E,F). However, ectopic CS formation in non-endodermal cells was not observed (Figure 4B,C,E,F). These results suggested that the initiation of CS formation in rice endodermal cells is likely to be related to high levels of *OsCASP1* expression. The underlying mechanism remains to be determined.

Previous studies have reported that Ca^2+^ enters the xylem radially via the apoplast in the root tip regions where CS is not completely formed or via the cytoplasm of non-suberized endodermal cells where the CS is formed [10]. In Arabidopsis, several CS mutants such as *esb1* and *lotr1* exhibited an enhanced suberization of endodermal cells, restricted Ca uptake, and showed low Ca accumulation in shoots [14,16]. Another CS mutant *sgn3-3* with no ectopic suberin accumulation also exhibited the lower Ca concentration in shoots, suggesting that CS is also important for Ca^2+^ transport into the xylem for transfer to the shoot [19]. In the present study, we found that the endodermal apoplastic barrier was developed early in the overexpressing lines (Figure 5A–F), which could be attributed to the early CS formation. However, the overexpression of *OsCASP1* did not affect suberin deposition in root endodermal cells (Figure 6A–F). Therefore, the decreased Ca accumulation in the roots and shoots of the *OsCASP1*-overexpressing lines was mainly caused by early formation of the endodermal apoplastic barrier. Early endodermal CS formation inhibited the apoplastic flow of Ca^2+^ across the endodermis into the xylem, resulting in less Ca uptake in the roots and less movement of Ca^2+^ to the shoots of the pOX lines. However, this inhibitory effect is not especially large because the effects of *OsCASP1* overexpression on Ca accumulation were observed only at high Ca concentrations. This is supported by the results from the short-term Sr uptake that *OsCASP1* overexpression does not affect apoplastic transport from root to shoot under normal conditions while reducing apoplastic transport with high Sr^2+^ treatment. Additionally, both apoplastic and symplastic pathways contribute to Ca accumulation in roots and shoots. There is one possibility that the contribution of the apoplastic pathway to Ca accumulation is higher in shoots than in roots. This may explain the smaller difference in Ca^2+^ concentrations between the WT and the pOX lines in roots compared to those in the shoots.

In conclusion, our results demonstrated that the overexpression of *OsCASP1* enhanced tolerance to high Ca stress in rice by decreasing the apoplastic flow of Ca^2+^ into the xylem and subsequently reducing Ca accumulation in the shoots. Our findings also provide the potential application of this strategy for improving the adaptation of other crops to high Ca environments, especially to a karst calcium-rich environment.

## 4. Materials and Methods

### 4.1. Generation of OsCASP1-Overexpressed Lines

For the overexpression of *OsCASP1* in rice, the *OsCASP1* coding region (Os04g0684300) was amplified by RT-PCR from rice total RNA using the primers 5’-AAAACTGCAGATGAGCTCCGGCGAGCCTGCCG-3’ (*PstI* site underlined) and 5’-CCGACGCGTCTAGCGCTTGCGGATAGAGCAG-3’ (*MluI* site underlined). Using *PstI* and *MluI*, the amplified fragment was cloned into the pCAMBIA1300-Ubi vector containing the maize ubiquitin promoter and the nopaline synthase gene terminator to form the Pubi-*OsCASP1* construct (Supplementary Figure S1). The resulting vector was introduced into *Agrobacterium tumefaciens* (strain EHA101) and then into rice (Nipponbare) by *Agrobacterium*-mediated transformation. Hygromycin was used to select putative transgenic plants. *OsCASP1* knockout line *oscasp1-1* was obtained by CRISPR/Cas9 technology and prepared before [18].

### 4.2. RNA Isolation and Gene Expression Analysis

To compare the expression of *OsCASP1* in the WT and transgenic plants, we extracted total RNA from the roots and shoots of both (5 days old) using the TRIzol reagent kit (Life Technologies, Carlsbad, CA, USA). First-strand cDNA was synthesized from 1 µg total RNA using the PrimeScript II 1st Strand cDNA Synthesis kit (Takara, Toyko, Japan) according to the manufacturer’s instructions. The SYBR Premix ExTaq II (Takara) was used for expression analysis by qTOWER 2.0 (Analytik Jena, Jena, Germany). The primer sequences for qRT-PCR were 5′-TCCCGGCCTTCCTGTTCTTC-3′ and 5′- CATGCATATGGCCACCCAGT-3′ for *OsCASP1*, 5′-GGTCAACTTGTTGATTCCCCTCT-3′ and 5′-AACCGCAAAATCCAAAGAACG-3′ for *Histone H3*. *Histone H3* was used as an internal standard. Three biological replicates were analyzed for each sample.

### 4.3. Determination of Ca Tolerance and Element Concentration

The WT and *OsCASP1*-overexpressing plants (15 days old) were grown in half-strength Kimura B solution containing different Ca^2+^ concentrations (0.18 and 20 mM) replacing the solution every two days. After 14 d, both control and experimental plants were washed three times in deionized water and photographed. The roots and shoots were collected separately and dried at 70 °C for 3 d before digestion with HNO_3_ at 140 °C. After appropriate dilution with 2% HNO_3_, the concentrations of zinc, manganese, iron, copper, potassium, magnesium, calcium, and strontium were measured by ICP-MS (Plasma Quant MS Elite; Analytik).

### 4.4. Xylem Sap Collection

Thirty-day-old seedlings were cultured for 6 h in half-strength Kimura B solution containing different Ca (0.18 and 10 mM) or Sr (0.18 and 20 mM) concentrations. The shoots (2 cm above the roots) were excised, and the xylem sap was collected using a micropipette for 1 h. After dilution with 2% HNO_3_, the Ca^2+^ concentration in the xylem sap was measured as described above.

### 4.5. Lignin/Cellulose Staining

To investigate the lignin deposition in the endodermis of the WT and *OsCASP1*-overexpressing plant roots, root cross-sections (100 µm thickness) were cut at several positions (1, 3, and 5 mm from the root apex) using a microslicer (VT1000 S; Leica, Germany). The sections were stained with 0.2% Basic Fuchsin for lignin and 0.1% Calcofluor White for cellulose as described previously [26]. Fluorescence was measured with a confocal laser scanning microscope (TCS SP8MP, Leica Microsystems). Z-stack magnified images were taken (20 optical sections with 0.3–0.4 µm intervals for each sample) and 3D images were constructed by LAS X 3D software (Leica Microsystems). The excitation and emission settings were 580 nm/600–615 nm for Basic Fuchsin and 405 nm/415–440 nm for Calcofluor White.

### 4.6. PI Penetration Assay

Propidium iodide (PI) staining was performed according to the method of Alassimone et al. [27]. Five-day-old seedling roots were treated with 15 µg/mL PI (P4170; Sigma Aldrick, St. Louis, MO, USA) in a dark incubator at 28 °C for 40 min. After three washes with water, the roots were sliced into 100 µm sections 15 and 30 mm from the root tip and evaluated by confocal microscopy using 488 nm and 600–650 nm as the excitation and emission wavelengths, respectively.

### 4.7. Suberin Staining

Suberin staining was performed as previously described [28]. Root cross-sections, prepared as described in 2.5, were stained with 0.01% (*w*/*v*) Fluorol Yellow 088 (Sigma) in lactic acid at 70 °C for 30 min. After washing three times at 70 °C, the specimens were observed under confocal microscopy. The following excitation and emission settings were used: 488 nm/510–525 nm.

### 4.8. Statistical Analysis

GraphPad Prism 8 software was used for statistical analysis. Data were analyzed using one-way ANOVA followed by Tukey’s test. Significance of differences at *p* < 0.05 is indicated by different letters.

## Data Availability

All the data supporting the conclusions of this article are provided within the article and in its additional files. All data and materials are available upon reasonable request from the corresponding author.

## Supplementary Materials

The following are available online at https://www.mdpi.com/article/10.3390/ijms22116002/s1.
