# Supplementary material for: Overexpression of OsCASP1 Improves Calcium Tolerance in Rice
Source: Int J Mol Sci. 2021 Jun 1;22(11):6002. doi: 10.3390/ijms22116002 (PMC8199569; doi:10.3390/ijms22116002)
Supplement: Supplementary file 1 [file ijms-22-06002-s001.zip › ijms-OXCASP1-Supplementary materials.pdf]

Supplementary Materials

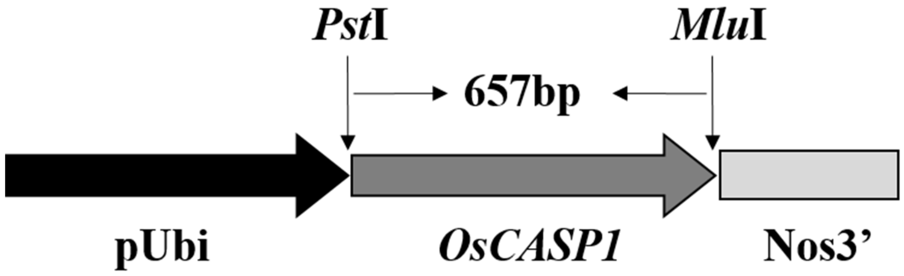

Supplementary Figure S1. Scheme of the construct used for the transformation. Maize Ubiquitin promoter was fused with *OsCASP1* cDNA (657 bp).

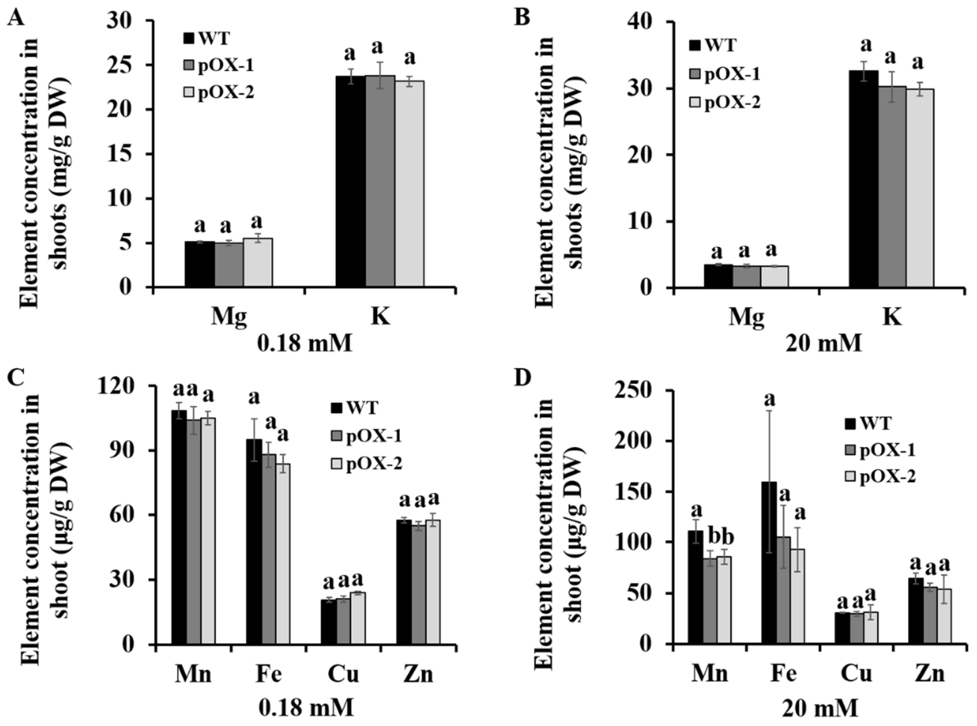

Supplementary Figure S2. Mineral elemental concentration in the shoots of wild-type rice and two *OsCASP1*-overexpressed lines at different Ca concentrations. (**A**) and (**B**) Concentration of Mg and K under different Ca conditions (0.18 and 20 mM). (**C**) and (**D**) Concentration of Mn, Fe, Cu and Zn under different Ca conditions (0.18 and 20 mM). Two-week-old seedlings were grown in a nutrient solution containing 0.18 or 20 mM $CaCl_2$ for 12 d. The data are presented as means ± SD (n = 3). Significant difference was determined by Tukey test and labeled with different letters ($p < 0.05$).

Fig. S3

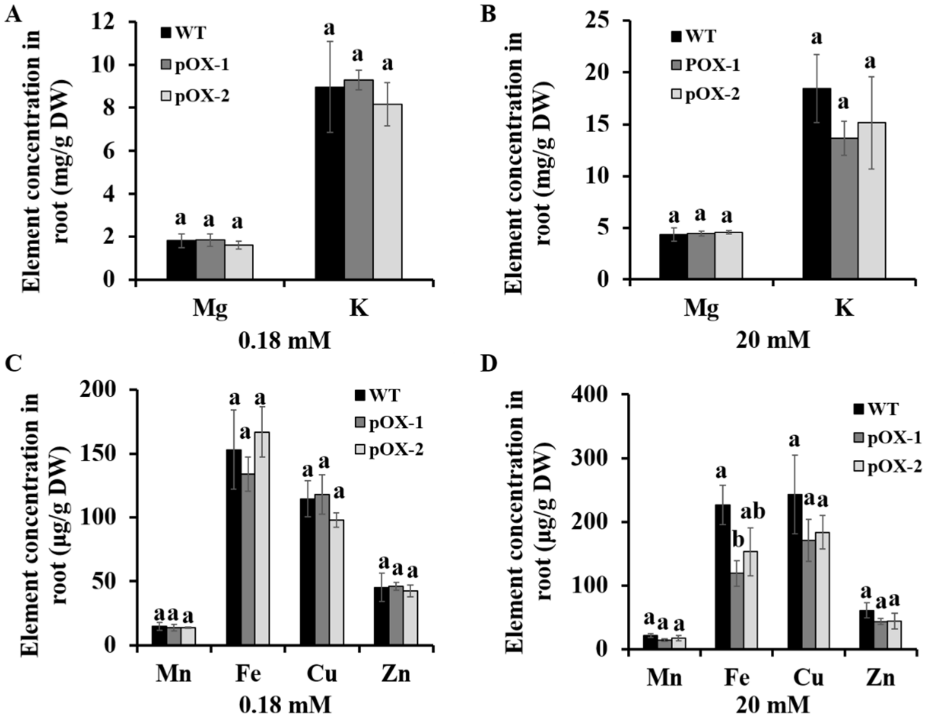

Supplementary Figure S3. Mineral elemental concentration in the roots of wild-type rice and two *OsCASP1*-overexpressed lines at different Ca concentrations. (**A**) and (**B**) Concentration of Mg and K under different Ca conditions (0.18 and 20 mM). (**C**) and (**D**) Concentration of Mn, Fe, Cu and Zn under different Ca conditions (0.18 and 20 mM). Two-week-old seedlings were grown in a nutrient solution containing 0.18 or 20 mM $CaCl_2$ for 12 d. The data are presented as means ± SD (n = 3). Significant difference was determined by Tukey test and labeled with different letters ($p < 0.05$).

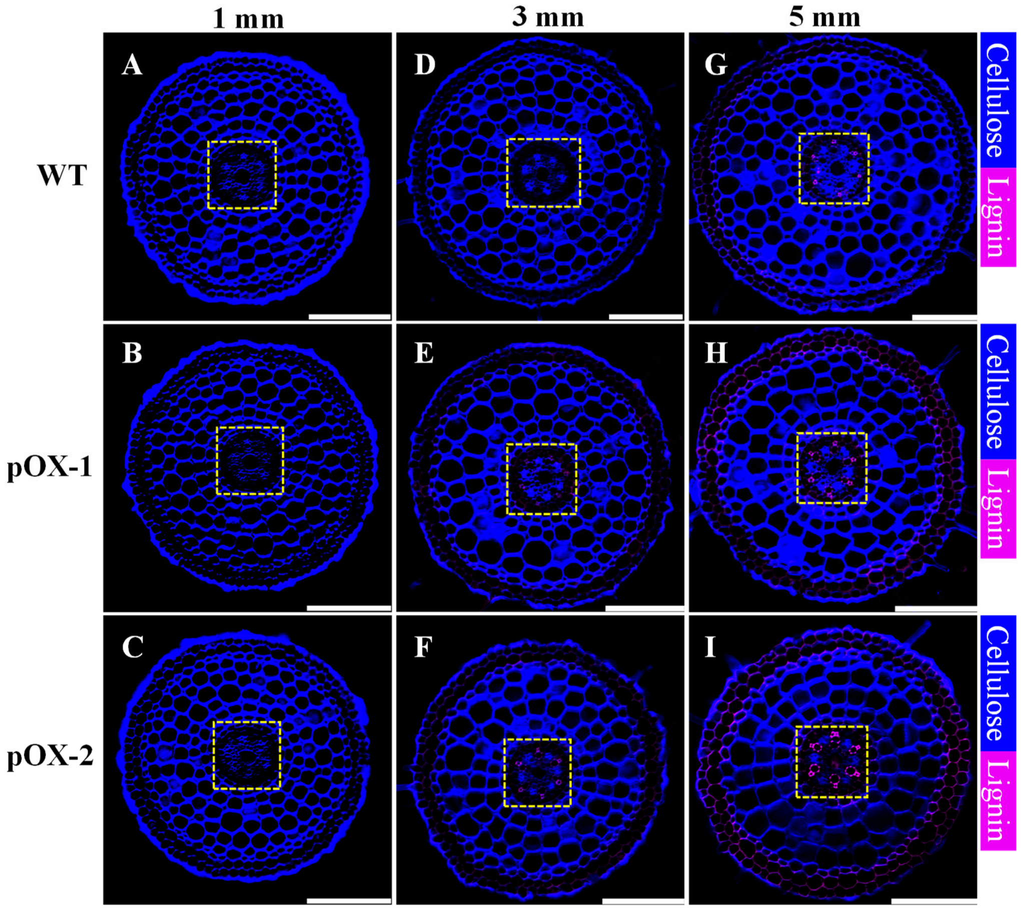

Supplementary Figure S4. Observation of CS formation in the wild-type plants and *OsCASP1*-overexpressed lines. (**A**) to (**I**) Observation of Casparian strip formation in the wild type (WT) and two *OsCASP1*-overexpressed lines. Lignin staining was performed in the root sections (1, 3, and 5 mm from the apex) of the wild type (**A**, **D**, **G**), pOX-1 (**B**, **E**, **H**) and pOX-2 (**C**, **F**, **I**). Magenta shows signal of lignin and blue shows cellulose. Scale bars, 100 μm. Image of the yellow box area in (**A**) to (**I**) was magnified and shown in Fig.4.
